# Carcass traits and meat quality of goats fed with cactus pear (*Opuntia ficus-indica* Mill) silage subjected to an intermittent water supply

**DOI:** 10.1038/s41598-022-25923-7

**Published:** 2023-01-16

**Authors:** Gabriel Ferreira de Lima Cruz, Edson Mauro Santos, Gherman Garcia Leal de Araújo, Paulo Sérgio de Azevedo, Ítalo Reneu Rosas de Albuquerque, Natália Matos Panosso, Alexandre Fernandes Perazzo, Anderson de Moura Zanine, Daniele de Jesus Ferreira, Anny Graycy Vasconcelos de Oliveira Lima, Juliana Silva de Oliveira

**Affiliations:** 1 Department of Animal Science, Federal University of Paraíba, Areia, Paraíba Brazil; 2 EMBRAPA Semiarid, Brazilian Agricultural Research Corporation, Petrolina, Pernambuco Brazil; 3 ADAPI, Piauí Agricultural Defense Agency, Teresina, Piauí Brazil; 4 Department of Animal Science, Universidade Federal do Maranhão, BR 222, km 4, s/n, Chapadinha, Maranhão 65500-000 Brazil

**Keywords:** Bacteria, Symbiosis, Carbohydrates, Enzymes, Biochemistry, Microbiology, Animal physiology, Environmental impact, Microbial ecology

## Abstract

The effect of different proportions of cactus pear (*Opuntia ficus-indica* Mill) silage (CPS) and intermittent water supply (IWS) to crossbreed goats' diets on carcass traits and meat quality were evaluated. The IWS caused a reduction (p = 0.03) in the percentage of leg fat in the animals. The rib eye area, carcass weight, and physical–chemical characteristics were not affected (p > 0.05) by the CPS or IWS. The IWS reduced (p = 0.04) the elongase enzyme activity. The CPS inclusion in the diet reduced C22:0 (p = 0.01), some branched-chain fatty acid (BCFA), C20:1 (p = 0.03), *c*13-C18:1 (p = 0.01) fatty acids. Therefore, in situations of water scarcity, an intermittent water supply of up to 48 h and diets with up to 42% cactus pear silage, can be adopted in goat feedlot, without affecting carcass traits and meat quality.

## Introduction

Semi-arid and arid regions around the world are home to large numbers of flocks of small ruminants subjected to water and feed shortages, which are increasingly intensified by the effects of climate change^1,2^. Livestock farming in these areas is greatly affected by long periods of drought and irregular rainfall, which lead to a low productive performance in animals due to the reduced amount of feed at specific times of the year. Alternatives that alleviate the difficulties of animal production in regions with low rainfall, irregular rainfall, and high evaporation, should be studied, in addition to the difficulty of accessing water sources^1^.

The intermittent supply of water is an alternative to mitigate water scarcity. Some previously published studies have shown that moderate water restriction does not result in considerable changes in the productivity and carcass and meat patterns of small ruminants^2,3^.

Another way to mitigate the effects of water scarcity is the availability of water through feed. The adoption of moist feeds in the diet of ruminants, among them the cactus pear containing a high-water content of 80–90%, minerals, and non-fiber carbohydrates (NFC) as energy source^4^.

Other authors observed the potential of diets based on cactus pear silage for animals in conditions of low water availability^3,4^, to meet the water needs of small ruminants, especially in the dry season. Some authors have observed an adequate fermentation profile of cactus pear silages^5,6^ and cite as advantages the possibility of increasing the frequency of harvesting cladodes, maximizing their use, and increasing total productivity per area, in addition to strategic use in greater amounts during periods of reduced availability of water and feed. Goats’ production in places with limited availability of water can encourage people to remain in arid places, given the lesser competition between goats and humans for water, through feeds such as cactus forage.

It is hypothesized that diets based on cactus pear silage and water supply intervals of up to 48 h do not affect the carcass parameters and meat quality of nondescript breed goats.

In view of the above, the objective was to evaluate the effect of intermittent water supply and the replacement of Tifton 85 grass hay (*Cynodon* sp.) with cactus pear silage on carcass traits, meat quality, fatty acid profile and nutraceutical parameters of crossbred goats finished in a feedlot.

## Results

### Morphometric measurements and carcass traits

There were no significant differences (*p* > 0.05) for most variables evaluated in morphometric measurements and carcass traits (Table 1), except for hot carcass yield (*p* < 0.001) and cold carcass yield (*p* < 0.001) which had lower averages for the animals that received 0% CPS.

**Table 1:** Morphometric measurements and carcass traits of goats fed with cactus pear silage (CPS) and submitted to intermittent water supply (IWS).

Item	CPS (% DM)			IWS (h)			SEM	*p-*value		
	0	21	42	0	24	48		CPS	IWS	CPS × IWS
Slaughter weight, kg	22.9	23.0	22.4	23.1	23.4	21.8	1.17	0.930	0.603	0.628
Body condition score	2.36	2.43	2.47	2.46	2.40	2.40	0.15	0.864	0.960	0.731
Body external length	51.5	52.8	52.6	52.4	52.3	52.2	0.78	0.438	0.983	0.234
Body internal length	59.3	59.5	59.5	59.3	59.9	59.1	0.96	0.975	0.827	0.598
Leg length	42.5	44.1	43.1	43.2	43.7	42.7	0.82	0.393	0.710	0.237
Thorax width	13.5	13.7	13.6	13.6	13.5	13.6	0.50	0.936	0.997	0.122
Rump width	17.5	18.3	16.8	17.4	18.0	17.3	0.73	0.369	0.755	0.854
Thoracic perimeter	59.3	61.2	59.7	60.0	60.6	59.5	1.14	0.479	0.792	0.648
Rump perimeter	49.1	51.2	49.9	49.4	50.9	49.9	1.39	0.564	0.747	0.716
Depth of chest	24.3	25.0	25.7	24.6	25.3	25.0	0.44	0.106	0.549	0.450
Carcass compactness index, kg/cm	0.16	0.17	0.17	0.17	0.17	0.16	0.01	0.464	0.866	0.401
Empty body weight, kg	17.8	18.8	18.5	18.6	18.7	17.8	0.99	0.744	0.785	0.592
Hot carcass weight, kg	9.67	10.4	10.4	10.3	10.4	9.71	0.56	0.582	0.632	0.451
Cold carcass weight, kg	9.53	10.2	10.2	10.2	10.3	9.57	0.56	0.595	0.634	0.451
Water evaporation losses in cooling, %	1.42	1.57	1.44	1.42	1.50	1.52	0.07	0.243	0.587	0.219
Hot carcass yield, %	42.1^b^	45.2^a^	46.2^a^	44.6	44.6	44.3	0.54	< 0.001	0.888	0.199
Cold carcass yield, %	41.7^b^	44.5^a^	45.6^a^	44.0	43.9	43.8	0.57	< 0.001	0.967	0.361
Biological yield, %	54.6	55.2	55.9	55.3	55.9	54.7	0.80	0.524	0.590	0.869
Measure C, mm	0.67	0.78	0.65	0.80	0.70	0.60	0.09	0.563	0.275	0.878
Loin eye area, cm^2^	8.00	7.60	7.40	7.90	7.35	7.80	0.39	0.513	0.436	0.195

^a,b^Means within rows with different letter superscripts differ by Tukey test (*p* < 0.05).

### Commercial cuts, tissue composition and leg muscularity index

There were no effects of diets, water supply or interaction between cactus pear silage and intermittent water supply (*p* > 0.05) to weights of the left cold carcass and the commercial cuts of goat meat (shoulder, neck, rib, leg, and loin) in kg or yield percentage (Table 2).

**Table 2:** Half cold carcass and weight of commercial cuts of goats fed with cactus pear silage (CPS) and submitted to intermittent water supply (IWS).

Item	CPS (% DM)			IWS (h)			SEM	*p*-value		
	0	21	42	0	24	48		CPS	IWS	CPS × IWS
Half carcass weight	5.29	5.28	5.01	5.23	5.37	4.99	1.04	0.759	0.654	0.370
**Commercial cuts, kg**										
Shoulder	1.04	1.06	0.99	1.06	1.05	0.99	0.05	0.586	0.586	0.380
Neck	0.54	0.51	0.48	0.51	0.52	0.50	0.03	0.505	0.903	0.422
Ribs	1.30	1.26	1.23	1.26	1.30	1.24	0.08	0.803	0.845	0.188
Leg	1.53	1.51	1.40	1.50	1.53	1.41	0.08	0.492	0.579	0.393
Loin	0.50	0.53	0.54	0.52	0.55	0.50	0.04	0.709	0.692	0.596
**Cuts yield, %**										
Shoulder	19.9	20.1	19.9	20.3	19.6	19.9	0.30	0.801	0.244	0.383
Neck	10.2	9.66	9.70	9.69	9.83	10.1	0.30	0.376	0.687	0.134
Ribs	24.6	23.9	24.5	24.1	24.1	24.8	0.54	0.639	0.552	0.438
Leg	29.0	28.6	28.2	28.7	28.8	28.3	0.46	0.464	0.754	0.516
Loin	10.1	10.1	9.86	10.0	10.2	9.86	0.32	0.847	0.832	0.850

When evaluating the tissue composition and the leg muscularity index (Table 3), we observed that the inclusion of cactus pear silage and/or the intermittent water supply did not present a significant difference between the treatments (*p* > 0.05), except for the leg fat percentage (*p* = 0.03), which was higher for the treatment without intermittent water supply. Leg fat (%) did not differ between diets with 24 and 48 h intermittent water supply.

**Table 3:** Tissue composition and leg muscularity index of goats fed with cactus pear silage (CPS) and submitted to intermittent water supply (IWS).

Item	CPS (% DM)			IWS (h)			SEM	*p-*value		
	0	21	42	0	24	48		CPS	IWS	CPS × IWS
**Leg weight, kg**	1.51	1.50	1.39	1.48	1.52	1.39	0.08	0.508	0.540	0.443
Muscle, kg	0.95	0.95	0.87	0.92	0.95	0.89	0.20	0.557	0.741	0.509
Fat, kg	0.17	0.15	0.13	0.18	0.14	0.13	0.02	0.166	0.078	0.856
Bone, kg	0.30	0.29	0.28	0.29	0.29	0.29	0.01	0.522	0.946	0.298
Other tissues, kg	0.07	0.08	0.07	0.07	0.08	0.07	0.02	0.787	0.113	0.109
Muscle:bone	3.15	3.26	3.13	3.18	3.28	3.08	0.12	0.719	0.505	0.621
Muscle:fat	6.43	6.93	7.55	5.96	7.45	7.55	0.67	0.494	0.187	0.206
Muscle, %	62.3	63.0	62.8	62.0	62.8	63.3	1.17	0.909	0.706	0.352
Fat, %	11.0	10.0	9.23	12.3^a^	8.80^b^	9.07^b^	0.96	0.451	0.032	0.361
Bone, %	20.2	19.4	20.2	19.5	19.3	21.0	0.61	0.638	0.122	0.787
Leg muscularity index	0.36	0.30	0.35	0.37	0.34	0.30	0.03	0.343	0.269	0.790

^a,b^Means within rows with different letter superscripts differ by Tukey test (*p* < 0.05).

### Physical–chemical characteristics of meat and chemical composition

Diets with cactus pear silage and intermittent water supply should be no differences (*p* > 0.05) for most variables of physical–chemical characteristics or chemical composition of meat (Table 4).

**Table 4:** Physicochemical composition of the *Longissimus lumborum* muscle of goats fed with cactus pear silage (CPS) and submitted to intermittent water supply (IWS).

Item	CPS (% DM)			IWS (h)			SEM	*p-*value		
	0	21	42	0	24	48		CPS	IWS	CPS × IWS
pH_initial_	6.56	6.62	6.59	6.56	6.54	6.66	0.06	0.799	0.355	0.873
pH_final_	5.73	5.76	5.73	5.68	5.87	5.68	0.11	0.989	0.394	0.629
**Color parameter**										
L* (lightness)	38.8	38.6	39.5	40.4	37.4	39.1	0.91	0.777	0.101	0.753
a* (redness)	16.8	17.1	17.3	16.6	17.4	17.2	0.61	0.839	0.627	0.579
b* (yellowness)	9.29	10.4	10.3	9.99	9.45	10.4	0.51	0.284	0.466	0.683
*Chroma* (saturation index)	19.2	20.0	20.2	19.4	19.9	20.1	0.71	0.616	0.791	0.650
Cooking losses, %	32.3	31.3	32.5	33.0	29.6	33.3	1.57	0.852	0.222	0.012
Shear force, N	20.7	19.5	19.9	19.3	19.3	21.6	0.11	0.785	0.290	0.018
**Chemical composition, %**										
Moisture	73.9	74.6	75.0	74.7	74.8	73.9	0.45	0.237	0.323	0.707
Protein	22.4	21.9	21.9	22.0	21.9	22.2	0.44	0.585	0.886	0.871
Total lipids	2.06	2.11	2.12	2.14	2.04	2.12	0.03	0.942	0.849	0.896
Ash	3.63^b^	3.79^b^	4.46^a^	3.99	3.88	3.98	0.11	< 0.001	0.739	0.398

^a,b^Means within rows with different letter superscripts differ by Tukey test (*p* < 0.05).

There was a significant interaction between cactus pear and the water supply to cooking losses (*p* = 0.012) and shear force (*p* = 0.018), in which animals that received a diet without cactus silage and without intermittent supply of water showed higher averages (Table 5).

**Table 5:** Interaction between cactus pear silage (CPS) and submitted to intermittent water supply (IWS).

Item	CPS (% DM)	IWS (h)		
		0	24	48
Cooking losses, %	0	38.22Aa	28.45Ba	30.37ABa
	21	25.68Bb	30.45ABa	37.53Aa
	42	34.99Aab	30.24Aa	32.14Aa
Shear force, N	0	23.98Aa	19.12Aa	18.93Aa
	21	16.48Bb	17.06Ba	24.42Aa
	42	17.46Aab	21.08Aa	21.08Aa

^A,B^^,a,b^Different letters within a row with uppercase and within a column with lowercase are significantly different by Tukey test (*p* < 0.05).

The ash content (*p* < 0.001) showed a significant difference with the inclusion of cactus pear silage and resulted in a greater proportion in the treatment with 42% inclusion. Treatments with 0 and 21% did not differ from each other.

### Lipid profile and nutraceutical parameters of meat

Intermittent water supply, cactus pear silage, and interaction between water supply and cactus pear silage did not influence (*p* > 0.05) most saturated fatty acids (SFA) present in the *Longissimus lumborum* muscle of the animals under study. The inclusion of cactus pear silage presented a significant difference between the treatments to docosanoic acid (C22:0, *p* = 0.009), branched-chain fatty acid total (∑ BCFA, *p* = 0.026), anteiso-tridecanoic acid (C13:0 anteiso, *p* = 0.026) and anteiso-pentadecanoic acid (C15:0 anteiso, *p* = 0.001) and resulted in a greater proportion in the treatment 0% CPS (Table 6). The tricosanoic acid (C23:0, *p* = 0.012) content differed between treatments of intermittent water supply.

**Table 6:** Fatty acid from *L. lumborum* muscle of goats fed with cactus pear silage (CPS) and submitted to intermittent water supply (IWS).

Variable (mg/g FAME)	CPS (% DM)			IWS (h)			SEM	*p-*value		
	0	21	42	0	24	48		CPS	IWS	CPS × IWS
∑ Saturated fatty acids	421	436	435	432	426	434	11.7	0.578	0.992	0.710
C6:0	0.20	0.15	0.15	0.15	0.19	0.15	0.02	0.207	0.083	0.533
C8:0	0.15	0.12	0.12	0.13	0.14	0.12	0.01	0.228	0.500	0.648
C10:0	0.96	0.88	1.01	0.95	0.95	0.95	0.05	0.920	0.770	0.903
C11:0	0.03	0.03	0.03	0.03	0.03	0.03	> 0.01	0.787	0.341	0.786
C12:0	0.49	0.44	0.46	0.46	0.47	0.47	0.02	0.487	0.993	0.572
C13:0	0.04	0.04	0.04	0.04	0.04	0.04	> 0.01	0.516	0.409	0.192
C14:0	13.4	13.3	14.4	13.5	13.2	14.4	0.01	0.444	0.159	0.227
C15:0	2.43	2.45	2.53	2.41	2.68	2.31	0.11	0.770	0.057	0.197
C16:0	228	230	230	225	222	241	5.49	0.939	0.055	0.284
C17:0	12.7	15.6	15.3	14.4	16.2	13.1	0.97	0.075	0.082	0.197
C18:0	154	166	163	168	161	154	8.70	0.533	0.556	0.452
C20:0	0.32	0.33	0.31	0.34	0.36	0.25	0.03	0.908	0.116	0.053
C21:0	0.00	0.06	0.04	0.03	0.06	0.02	0.03	0.299	0.631	0.669
C22:0	1.10^a^	0.76^ab^	0.52^b^	0.87	0.89	0.63	0.01	0.009	0.289	0.403
C23:0	0.21	0.18	0.17	0.13^b^	0.26^a^	0.18^ab^	0.03	0.767	0.012	0.065
C24:0	0.04	0.00	0.03	0.01	0.05	0.03	0.02	0.553	0.428	0.301
∑ Branched-chain fatty acid	7.15^a^	6.02^b^	6.25^b^	6.68	6.51	6.21	0.27	0.026	0.260	0.688
C13:0 *anteiso*	0.51^a^	0.34^b^	0.31^b^	0.39	0.44	0.33	0.05	0.026	0.051	0.683
C14:0 *iso*	0.20	0.15	0.17	0.18	0.18	0.17	0.01	0.103	0.751	0.446
C15:0 *iso*	0.85	0.72	0.81	0.82	0.76	0.80	0.05	0.205	0.675	0.250
C15:0 *anteiso*	1.07^a^	0.78^b^	0.83^b^	0.90	0.93	0.85	0.06	0.001	0.379	0.155
C16:0 *iso*	1.27	1.01	1.07	1.23	1.07	1.04	0.07	0.069	0.122	0.926
C17:0 *iso*	3.25	3.01	3.05	3.16	3.13	3.02	0.14	0.696	0.716	0.638

^a,b^Means within rows with different letter superscripts differ by Tukey test (*p* < 0.05).

Among the monounsaturated fatty acids (MUFA), only the C20:1 and the c13C-18:1 showed a significant difference with the inclusion of cactus pear silage, showing greater averages for the treatment with 0% cactus pear silage inclusion (Table 7).

**Table 7:** Monounsaturated fatty acids (MUFA) from *Longissimus lumborum* muscle of goats fed with cactus pear silage (CPS) and submitted to intermittent water supply (IWS).

Variable (mg/g FAME)	CPS (% DM)			IWS (h)			SEM	*p-*value		
	0	21	42	0	24	48		CPS	IWS	CPS × IWS
∑MUFA	528	521	523	524	528	520	11.9	0.814	0.990	0.670
C10:1	0.01	0.01	0.01	0.01	0.01	0.01	> 0.01	0.920	0.770	0.903
C17:1	8.91	9.05	9.21	8.99	9.90	8.27	0.51	0.949	0.177	0.351
C20:1	1.20^a^	0.90^b^	1.16^ab^	1.14	1.08	1.04	0.07	0.026	0.689	0.286
C22:1 n-9	4.62	3.76	4.02	4.00	4.57	3.83	0.35	0.178	0.173	0.173
C24:1	1.52	1.44	1.43	1.56	1.43	1.39	0.09	0.866	0.246	0.222
Σ*cis*-MUFA	506	500	501	502	505	500	11.7	0.839	0.989	0.709
*c*9-C14:1	0.73	0.64	0.63	0.60	0.68	0.73	0.05	0.370	0.223	0.083
*c*9-C16:1	23.7	21.5	21.5	21.4	22.0	23.3	1.22	0.315	0.484	0.261
*c*9-C18:1	413	422	422	417	424	416	9.82	0.933	0.985	0.880
*c*11-C18:1	41.2	32.8	33.4	37.4	34.6	35.4	2.43	0.051	0.563	0.735
*c*12-C18:1	17.1	15.2	15.1	16.0	15.6	15.7	0.97	0.295	0.866	0.616
*c*13-C18:1	9.32^a^	7.62^b^	7.70^b^	8.62	7.96	8.06	0.42	0.011	0.263	0.449
*c*15-C18:1	0.64	0.61	0.67	0.64	0.59	0.71	0.08	0.779	0.638	0.197
Σ*trans*-MUFA	6.00	5.80	6.00	6.20	5.40	6.10	0.45	0.880	0.393	0.173
*trans-*C18:1	2.71	2.63	2.61	2.99	2.40	2.56	0.26	0.993	0.361	0.387
*t*16-C18:1	3.34	3.13	3.39	3.25	3.03	3.59	0.31	0.735	0.190	0.109

^a,b^Means within rows with different letter superscripts differ by Tukey test (*p* < 0.05).

Cactus pear inclusion or the intermittent water supply did not provide significant differences (*p* > 0.05) for polyunsaturated fatty acids (PUFA) (Table 8) or fatty acid ratio or nutraceutical parameters on goats' meat (Table 9), except to the elongase that showed greater activity (*p* = 0.045) in the meat of the animals that were submitted to 24 h of intermittent water supply.

**Table 8:** Polyunsaturated fatty acids (PUFA) from *Longissimus lumborum* muscle of goats fed with cactus pear silage (CPS) and submitted to intermittent water supply (IWS).

Variable (mg/g FAME)	CPS (% DM)			IWS (h)			SEM	*p-*value		
	0	21	42	0	24	48		CPS	IWS	CPS × IWS
∑PUFA	50.7	43.2	42.3	44.2	46.5	45.4	3.12	0.146	0.782	0.082
c9,t11-C18:2 + isomers	0.69	0.81	0.74	0.80	0.67	0.77	0.09	0.645	0.464	0.334
∑n-3	10.1	7.5	6.73	7.67	8.67	7.97	1.08	0.125	0.450	0.162
C18:3 n-3	0.88	0.52	0.78	0.62	0.69	0.88	0.10	0.072	0.190	0.809
C20:3 n-3	0.03	0.08	0.06	0.05	0.06	0.05	0.02	0.139	0.740	0.296
C20:5 n-3	3.00	2.41	2.08	2.27	2.79	2.43	0.43	0.438	0.458	0.603
C22:5 n-3	4.95	4.04	3.51	3.87	4.66	3.98	0.64	0.409	0.261	0.143
C22:6 n-3	1.21	0.45	0.30	0.86	0.47	0.63	0.21	0.061	0.588	0.676
∑n-6	40.1	34.9	34.8	35.8	37.3	36.6	2.39	0.261	0.937	0.132
c9,c12-C18:2 n-6	20.1	19.3	19.9	19.0	20.3	20.0	1.27	0.800	0.899	0.437
C18:3 n-6	0.20	0.23	0.22	0.23	0.21	0.22	0.03	0.805	0.959	0.900
C20:2 n-6	0.01	0.00	0.03	0.00	0.02	0.02	0.01	0.189	0.619	0.505
C20:3 n-6	0.45	0.48	0.43	0.43	0.55	0.38	0.08	0.816	0.055	0.056
C20:4 n-6	19.3	14.9	14.2	16.1	16.2	16.0	1.55	0.085	0.968	0.215

^a,b^Means within rows with different letter superscripts differ by Tukey test (*p* < 0.05).

**Table 9:** Fatty acid ratio and nutraceutical parameters from *Longissimus lumborum* muscle of goats fed with cactus pear silage (CPS) and submitted to intermittent water supply (IWS).

Variable (mg/g FAME)	CPS (% DM)			IWS (h)			SEM	*p-*value		
	0	21	42	0	24	48		CPS	IWS	CPS × IWS
∑PUFA: ∑SFA^1^	0.12	0.10	0.10	0.10	0.11	0.10	0.01	0.084	0.825	0.108
∑PUFA ∑MUFA^2^	0.10	0.08	0.08	0.08	0.09	0.09	0.01	0.344	0.770	0.106
∑MUFA: ∑SFA^3^	1.25	1.19	1.20	1.21	1.24	1.20	0.06	0.720	0.983	0.698
n-6/n-3	3.98	4.65	5.17	4.66	4.30	4.59	0.59	0.267	0.272	0.407
Desirable fatty acids	732	730	729	735	736	720	5.75	0.854	0.124	0.309
Atherogenicity index	0.29	0.29	0.29	0.29	0.28	0.31	0.01	0.864	0.295	0.529
Thrombogenicity index	0.79	0.83	0.82	0.82	0.79	0.83	0.04	0.686	0.976	0.781
h:H index^4^	1.91	1.92	1.89	1.94	1.98	1.79	0.08	0.969	0.351	0.672
Δ9-dessaturase C16	9.38	8.58	8.55	8.75	8.97	8.81	0.48	0.389	0.992	0.681
Δ9-dessaturase C18	72.9	71.7	72.1	71.3	72.5	72.9	1.47	0.772	0.732	0.563
Elongase	69.2	70.1	70.0	70.4^ab^	70.6^a^	68.3^b^	0.67	0.661	0.045	0.221

^a,b^Means within rows with different letter superscripts differ by Tukey test (*p* < 0.05).

^1^Ratio of polyunsaturated fatty acids to saturated fatty acids.

^2^Ratio of polyunsaturated fatty acids to monounsaturated fatty acids.

^3^Ratio of monounsaturated fatty acids to saturated fatty acids.

^4^Ratio of hypocholesterolemic to hypercholesterolemic fatty acids.

## Discussion

Morphometric measurements are subjective and used to assess the carcass development and quantitatively measure the muscular distribution in the carcass with estimates of its conformation. In the present study there were not significative differences observed for these parameters or for carcass compactness index (CCI), inferring that the use of cactus pear silage as well as intermittent water supply combined or alone did not alter animal growth and/or carcass conformation, maintaining the muscle pattern achieved by the control diet (usual) and demonstrating body and carcass uniformity. Since animals used in this study were homogeneous and had similar age and body performance, as indicated by the carcass morphometric measurements and by the difference between the empty carcass and hot carcass weights, which resulted in the sum of head + limb with an average of 8.2 ± 0.13 kg between treatments, giving an idea that the animals were similar in chronological age, since the allometric growth of the body occurs from the extremities to the interior of the body.

The significant difference between treatments with inclusion of cactus pear silage for hot carcass yield (HCY) and cold carcass yield (CCY) may be related to the weight of the full gastrointestinal tract, which showed higher values for animals fed with a higher proportion of Tifton 85 grass hay in the diet (0% CPS). Increasing the NDF content of the diet reduces the passage rate of digesta, and the emptying of the gastrointestinal tract (GT) that cause a distension of the rumen-reticulum and increase the weight of the gastrointestinal tract, resulting in lower HCY and consequently lower CCY. While the diets with inclusion of CPS increase NFC content, such as pectin, which have higher rates of rumen degradability and, higher rates of passage^7–9^.

Measurements and evaluations carried out on the carcass, such as the carcass compactness index and loin eye area (LEA), are parameters that quantitatively measure the muscle distribution in the carcass, an edible part of greater financial return, which indicates the conformation of these animals^3^, while the body condition score (BCS) and the measure C, which are highly correlated, measure the distribution of fat on the carcass, giving an idea of the carcass finish, in which the higher these variables, the greater the proportion of fat that allows for less water loss due to carcass cooling^10^. These variables in the present study were also not influenced by the levels of cactus pear silage and water restrictions, presenting an overall mean of 0.17 kg/cm, 7.68 cm, 2.42 points and 0.7 mm respectively, and consequently did not influence the losses due to cooling, which presented an average loss of 1.48%.

The main cuts of the goat carcass are the neck, leg, shoulder, loin, and rib. Their economic values differ, and their proportions become an important index to evaluate the carcass quality^9^. The cuts of greatest importance and commercial values are the leg and the loin, called noble cuts because they present greater yield and muscle tenderness, being interesting that they present a good proportion in the carcass, for providing greater edible tissue content, mainly muscle.

Carcasses with similar weight tend to have equivalent proportions of cuts, as they exhibit isogonic growth. As the cold carcass weight (CCW) and the conformation of the animals were similar, with similar morphometric measurements, they had a direct relationship in the absence of an effect on commercial cuts.

The commercial value of the carcass, whether through carcass yield and/or the proportions of the cuts, is also linked to tissue composition, thus the dissection of the leg represents an estimate of measuring the tissue composition of the carcass, in which is sought a greater proportion of muscle, intermediate proportion of fat and less bone in carcasses^11^. In this way, diets with cactus pear silage and the different levels of intermittent water supply resulted in the constancy in the amount of muscle, fat, and bone in legs of goats. The similarity in muscle proportion is related to the lack of effects on slaughter weight and CCW, as the weight of muscles is highly correlated to carcass weight. The average muscle yield was above 60% in all treatments, confirming that the animals showed good efficiency to the diets and adapted well to the water supply levels. Although the diets with cactus silage had high amounts of metabolizable energy (ME) and no difference in DM intake, the energy input was similar that not influencing carcass weights and carcass compactness index. That is, it did not influence muscle deposition in the carcass, probably due to synchronicity of energy and protein.

As for the weight and proportion of bone tissue, it is believed that because this is a tissue with early development in relation to muscle and fat^2^, diets in the final stages of growth (average of 8 months) would hardly change their participation in the tissue composition, where the relationship of this tissue with the others is usually only increased when there are changes in the proportion of muscle and/or fat.

Water restriction, as long as it is moderate and acute, mainly affects the loss of body water and not tissues, which does not cause deleterious effects on animal productivity and growth.

The muscle:fat ratio indicates the state of leg fattening, while the muscle:bone ratio estimates the carcass muscularity, both being attributes of quality^3^. The similarity previously reported in the weight of fat, bone and muscle corroborates that these relationships also do not have differences. The same occurs for the leg muscularity index (LMI), due to the weight of the five muscles used to determine the index and the length of the femur which had been similar between the animals.

Nevertheless, when considering fat as a percentage of participation in leg weight, it is possible to observe that the intermittency in water supply in both intervals (24 and 48 h) reduced the proportion of fat in the leg. Although in this research, the water supply levels did not affect the daily intake of dry matter from animals, with average intake of 650.67 g/kg DM, ranging from 599 to 682 g/kg DM between treatments^7^, during days of water deprivation, fat mobilization for energy availability may occur, possibly offsetting water stress and influencing not only feed intake, on these days of deprivation but also affecting energy metabolism, which results in the mobilization of energy reserves^2^.

When the physicochemical composition of the meat was evaluated, it was observed that the diets and water supply levels probably did not affect the reserves of muscle glycogen during the pre-slaughter management as can be seen through pH_initial_ and pH_final_. The pH_initial_ right after slaughter should be close to neutrality, as well as in the live animal, indicating that the animal did not suffer from stress during the pre-slaughter period. The pH_final_, on the other hand, is expected to show a considerable variation, between 5.55 and 6.2 for goat meat; and due be inversely proportional to the concentration of muscle glycogen at the time of slaughter, that is, a more intense expenditure of glycogen stores results in less lactic acid production and higher pH_final_^10,12,13^. In this research, the pH_final_ had an average of 5.74, a pH higher than the isoelectric point of muscle proteins (5.2–5.3). This result is favorable, since it is above the neutral charge and presenting an excessive negative charge that provides the repulsion of filaments, which allows water molecules to bind and improve the organoleptic characteristics of the meat, through succulence and texture of meat^13^ evaluated by cooking loss, moisture, and shear force, principally. The cooking loss (CL), moisture and shear force (SF) were within the values recommended (20–35% CL, moisture above 70% and SF up to 44.13 Newton (N) for goat meat) to classify the meat as soft and tender^14^. Statistically, interactions were found between the supply of silage and intermittent water supply, in which goats on a diet without cactus pear silage and without intermittent water supply showed higher values of cooking losses and shear force.

Higher concentrations of collagen content and/or greater activities of calpastatin (which inhibit the action of calpains), as well as larger fascicles and greater number of fibers present in each muscle fascicle, as was visually observed in the meat of the animals in this research, can lead to reductions in meat tenderness^15^. Because goat carcasses are generally small, with low marbling degree and a thin layer of subcutaneous fat, there is rapid heat dissipation at the beginning of the post-mortem period, which can lead to cold shortening, muscle hardening, and less tender meats^16^.

pH_final_ of the meat has a high correlation with color parameters (L*—lightness, a*—redness, b*—yellowness and *Chroma*), as the pH_final_ can affect the reaction of myoglobin to oxymyoglobin. The b* index in meat, on the other hand, may be related to the concentration of fat and/or the presence of carotenoids in the diet which can be affected by forage preservation processes, such as silage and hay, which significantly reduces by up to 80% carotenoids levels^13^. It is believed that the carotenoid concentrations in the diet of this study were similar between treatments and consequently in values of b* of meat. Values of a* and *Chroma* directly depend on the content and state of the heme pigments in the muscle, due to the chemical state of iron (Fe), playing an important role in meat color^10^. These parameters showed no significant difference between treatments, however, higher values of a* and *Chroma* in meat are desired, as a result of the increase in oxymyoglobin and decrease in metmyoglobin that provides the meat's "bloom". According to Dawson et al*.*^17^, the minimum critical value for meat luminosity (L*) is 34. Lower values of L are related to elevating pH_final_, which results in the high concentration of metmyoglobin, making the meat darker, which causes rejection by consumers for associating dark meat to as old meat.

The meat's presentation and more precisely its color is an important factor that can influence a consumer’s purchase decision, as it gives us the idea of freshness and meat' quality. The L* and a* color parameters are the most representative for these characteristics^18^. Although in our research it did not have a significant effect on the color parameters, we can indicate that the meat obtained in this research would be well accepted by consumers, because Hopkins^19^ suggests that consumers will consider meat color acceptable when the L* value is equal to or exceeds 34, and a* value below 19 or equal to or exceeds 9.5 according to Khliji et al*.*^18^. In the present study, all values for L* remained above this aforementioned threshold and the values of a* remained within these values which suggests that meats from all diets and water supply levels had an acceptable color for consumers.

When evaluating the chemical composition of meat, no significant differences were observed between treatments, except for the ash content, that remained above the average values found in the literature, which is 0.99–1.10%^16^. It is believed that because cactus pear is a rich source of Ca, Mg, K and with increasing level of cactus pear silage in the diet^31^, these minerals were consumed in larger amounts, which could have resulted in a higher proportion of minerals in the meat of animals that received 42% cactus pear silage.

The lipid fatty acid profile in meat has a major impact on sensory properties and nutritional quality, influencing acceptance and health for consumers^20,21^. Intermittent water supply, cactus pear silage, and interaction between water supply and cactus pear silage did not influence most fatty acids present in the *Longissimus lumborum* muscle of the animals under study, except only a few saturated fatty acids e.g. docosanoic acid (C22:0), tricosanoic acid (C23:0), BCFA, anteiso-tridecanoic acid (C13:0 anteiso) and anteiso-pentadecanoic acid (C15:0 anteiso).

Biohydrogenation of ruminal bacteria results in a circumstantial variety of fatty acids (FA), which will be absorbed in the intestine and later incorporated into the meat of goats. In addition to the diet and the biohydrogenation, the meat lipid profile can vary due to de novo synthesis, desaturation, duration of the feeding period and differences in pathways of various FA by the animal organism^22^.

A high concentration of saturated fatty acids present in meat is not desirable, as there is evidence that saturated fatty acids, mainly C16:0, as well as myristic (C14:0) and lauric (C12:0) increase the blood cholesterol and low-density lipoproteins (LDL) concentration, due to interferences with hepatic LDL receptors^23^, however, in the studied treatments, there were no significant differences for these fatty acids. On the other hand, C18:0 has no impact on cholesterol levels, due to being poorly digested and easily desaturated to C18:1 by Δ9-desaturase^24^, present in the cell endoplasmic reticulum. This fatty acid is not harmful to health and is considered the only desirable SFA. As the levels of C18:0 in diets tend to be minimal, their main origin is the biohydrogenation of PUFA and de novo syntheses in diets with a high energy pattern^25^.

In addition to carrying out the biohydrogenation process, ruminal bacteria synthesize a series of FA, mainly those of odd and branched chain, that comprise mainly the lipids of the bacterial membrane^26,27^, to maintain membrane fluidity. Linear odd-chains fatty acids are formed when propionyl-CoA, instead of acetyl-CoA, is used as a de novo synthesis initiator^25^. On the other hand, iso and anteiso FA are synthesized by the precursors branched-chain amino acids (valine, leucine, and isoleucine) and their corresponding branched- short-chain carboxylic acids (isobutyric, isovaleric and 2-methyl butyric acids)^28^.

There is an increasing interest to study odd-and branched-chain fatty acids (OBCFAs) from animal products, mainly in milk due to its higher concentration compared to meat. Researchers reported that several OBCFAs have potential health benefits in humans^29^ as improved gut health^30^ and presenting anti-cancer activity^31^, as well as improve the sensory characteristics of the meat, providing a greater sensation of tenderness and juiciness, because BCFA content are associated with a less consistent fat in meat from lambs due to its lower melting point and its chain structure^32^.

The FAs profile in the ruminal bacteria is largely composed by OBCFAs (C15:0; anteiso C15:0; iso C15:0; C17:0; iso C17:0; C17:1 and anteiso C17:0) in the bacteria membrane lipids^24^. Thus, the higher concentration of OBCFAs might be the result of the difference in the rumen bacterial populations induced by variation in the dietary carbohydrate, that is, a higher concentration of cellulolytic bacteria in relation to amylolytic bacteria, due to the high neutral detergent fiber (NDF) content in the diet with 0% cactus forage silage. It is also known that amylolytic bacteria produce more linear odd chain and anteiso FAs than iso FAs, whereas cellulolytic bacteria produce more iso FAs^28,32^. As the Tifton 85 grass hay-based diet had the highest neutral detergent fiber corrected for ash and protein (NDFap) and starch content (highest % of ground corn), the meat of those animals had higher concentrations of anteiso C15:0 and anteiso C13:0 compared to animals fed diets with the inclusion of cactus pear silage, also influencing the total sum of branched chain fatty acids.

Although levels of intermittent water supply have generated punctual changes in tricosanoic acid (C23:0) SFA, the same was not observed for MUFA and PUFA, due to changes in the rumen environment, promoted by water restrictions, which were not sufficient to circumstantially modify biohydrogenation, resulting in similarities in concentrations of unsaturated fatty acids in goat meat.

The animals subjected to 24 h of intermittent water supply (IWS) presented the highest concentration of C23:0 in relation to other treatments, which is interesting because it is involved in the synthesis of ceramide and reduces the risk of diabetes in humans^33^.

The cactus pear has high non-fibrous carbohydrate (NFC) content (mainly pectin), having 59.5% high and medium rumen degradation carbohydrates which provide a higher production rate and removal of short-chain fatty acids and changes in rumen bacterial populations^34^. The inclusion of CPS resulted in a higher passage rate of digesta, affected biohydrogenation, and resulted in the escape of intermediate fatty acids isomers that are absorbed in the small intestine. Consequently, there was changing composition of fatty acids in the muscle of these animals, with a significant effect being observed only in the cis-13 C18:1. Furthermore, diets with high proportions of cactus pear silage (CPS), such as 42% CPS diet, can decrease ruminal pH and affect the final stages of biohydrogenation, resulting in the escape of intermediate fatty acids isomers, that are absorbed in the small intestine, which can explain the similarity of the C20:1 in 42% CPS diet from the Tifton hay-based diet, with differences between goat meat from 21% CPS diet and Tifton hay-based diet.

Oleic acid (*c*9-C18:1) was the MUFA with the highest participation in the lipid profile of goat meat, which is interesting because it has a hypocholesterolemic effect, being a desirable fatty acid (DFA) for not reducing the serum high density lipoproteins (HDL) levels and thus prevent cardiovascular disease by reducing LDL levels^35^. The high concentrations of *c*9-C18:1 in ruminant meat come from the food intake, the effect of biohydrogenation, and mainly of the high activity of Δ9-desaturase, necessary for animal biosynthesis through desaturation of C18:0 to *c*9-C18:1^27^. This fatty acid in the lipid profile of red meat varies between 30 and 43%^36^, confirming that the meat in the present study had a good concentration of this fatty acid.

Much of unsaturated fatty acids, which have 18 carbons or 16 carbons, are largely converted to C18:0 and C16:0 through biohydrogenation, and when this process is not 100% completed, in addition to the PUFA that pass through this process intact, some product intermediates are formed, reaching the duodenum and are absorbed by the animal, in which significant amounts of cis and trans-monounsaturated, such as vaccenic fatty acid (*t*11-C18:1), reach the duodenum and are absorbed, later composing the muscle tissue^22^.

The literature indicates that the precursor of conjugated linoleic acid (CLA) in the meat of animals is trans vaccenic acid (*t*11-C18:1), so the enzyme ∆9-desaturase, besides acting in the conversion of stearic into oleic fatty acid, also converts the trans-vaccenic acid to its corresponding CLA isomer, *c*9*t*11-C18:2^36^. This pathway is more expressive in the mammary gland, and as the concentration of vaccenic acid (*t*11-C18:1) was not different, the concentration of CLA was not affected by the supply of silage and intermittent water supply, in the same way, that there are also no differences in the activity of ∆9-desaturase. Nevertheless, it is worth noting that in the human adipose tissue there is also the presence of ∆9-desaturase, and therefore, increased intake of vaccenic fatty acid could have the same beneficial effects associated with the intake of CLA, where the dietary vaccenic fatty acid shows 19–30% conversion rate^37^.

Tifton hay is a natural source of n-3 fatty acids, mainly C18:3 n-3 with up to 20% participation in the lipid profile^2^, allowing a certain part of these PUFAs to be absorbed and increased in the tissue muscle, with 10 to 30% PUFAs in the diet generally escaping from biohydrogenation.

Linoleic fatty acid (*c*9*c*12 C18:2) and α-linolenic acid (C18:3 n-3) are essential fatty acids for humans, that serve as precursors of the n-3 and n-6 pathways, distinct families, but synthesized by some of the same enzymes (∆4-desaturase, ∆5-desaturase, and ∆6-desaturase)^25^. Arachidonic fatty acid (C20:4 n-6) comes from elongation and desaturation of linoleic acid, where its concentrations, even close to that of its precursor, may indicate that there was a high activity of ∆6-desaturase (desaturation to γ-linolenic), elongase (elongation of γ-linolenic to dihomo-gamma-linolenic) and ∆5-desaturase. This fatty acid was influenced by the diets, presenting lower concentrations in the meat of animals fed the 42% cactus pear silage when compared to the Tifton hay diet (0% cactus pear silage).

A higher concentration of long-chain PUFA n-3, docosahexaenoic (C22:6 n-3), was observed in the muscle of animals fed on Tifton hay. This was probably due to the high concentration of C18:3 n-3, precursor of C22:6 n-3, that the hay presents in relation to the cactus pear silage.

The ratios and proportions of fatty acids are used to determine nutritional and nutraceutical values of the product or diet, and mainly, to indicate the cholesterolemic potential^4^. It is interesting that the n-6/n-3 ratio is low due to the pro-inflammatory properties of n-6; it is recommended to decrease its intake to assist in disease prevention^38^, while n-3 fatty acids are anti-inflammatory, antithrombotic, antiarrhythmic and reduce blood lipids, with vasodilating properties, being interesting that they present a higher proportion^24^. n-6 fatty acids tend to have a higher percentage in meat, and this directly influences the formation of n-3 isomers, since linoleic acid, when in excess, can reduce the synthesis of linolenic acid metabolites. The percentage of FA in one group can interfere with the metabolism of the other, reducing its incorporation into tissue lipids and altering its general biological effects^38^. Therefore, it is not recommended that the n-6/n-3 ratio be kept above 5 or 6^39^, demonstrating that the averages of the current research remained acceptable.

In relation to atherogenicity index (AI) and thrombogenicity index (TI), Ulbricht and Southgate^39^ proposed that sheep meat should have values of up to 1.0 and 1.58, respectively, and the lower the values for these indices in the lipid fraction, the greater the prevention of early stages of cardiovascular diseases. In the present study, the general averages observed were 0.29 for the AI, and 0.81 for the TI, although there were no significant differences, all treatments are within the recommended range, despite having been used as comparative standard to sheep, due to the absence of the proposed standard for goat meat.

The h:H ratio did not differ for diets and water supply levels, but had an average of 1.90, below the reference value for meat products, which is 2.0. Values above 2.0 are recommended and favorable^40^, as it indicates a higher proportion of hypocholesterolemic fatty acids, that are beneficial to human health.

The ∆9-desaturase enzyme that acts on both the mammary gland and adipose tissue, responsible for the transformation of SFA into unsaturated fatty acids (UFA), as well as in the endogenous conversion of CLA^37^ did not differ between treatments. On the other hand, the elongase showed less activity. Probably there was a greater "de novo" synthesis which resulted in a greater accumulation of palmitic fatty acid, and a reduction in the activity of the elongase enzyme.

The crossbred goats demonstrated to present efficient mechanisms for adapting to water restrictions, especially when receiving feed with higher water content, such as cactus pear silage, being able to replace Tifton hay with 42% cactus pear silage in the diet for goats in confinement without negatively affecting the carcass traits and meat quality. Because, although these animals have shown some differences in the indices of tenderness and juiciness of their meats, however, all presented values of juiciness and tenderness compatible with meat extremely appreciated by the consumer market, and even goat meat showing some fatty acids with different concentrations induced by the supply of silage and water intermittence, the final lipid profile was appropriate to the health of consumers, observed by the absence of differences in the total concentrations of PUFA and in the main nutraceutical parameters (DFA, n-6/n-3; h:H; AI and TI).

These results are relevant, indicating that goat feedlots in regions with low water availability may adopt strategies of lesser demand for drinking water and considerable concentrations of cactus pear silage in the diet, can reduce production costs without considerably affecting the product to be marketed, and therefore, provide higher profitability of the system.

## Methods

The study was conducted from December 2014 to February 2015 in the Animal Nutrition sector of the Brazilian Agricultural Research Corporation—Semi-arid EMBRAPA, located in the municipality of Petrolina, state of Pernambuco, Brazil, at 9°23′35″ S and 40°30′27″ W. Animal care and standards used were approved and based on the National Council for the Control of Animal Experimentation the Federal University of Bahia (Protocol 04/2016—IMS/CAT—UFBA). All the research was performed in accordance with relevant guidelines and regulations. The Semi-arid EMBRAPA was the supplier of cactus pear (*Opuntia ficus indica* Mill), thus all local guidelines were adhered to for the use of plants and its use for silage production. Study design, animal experiments, and reporting followed the ARRIVE guidelines.

### Animals experimental design, and duration

Thirty-six castrated male crossbreed goats (F1 Boer × mixed breed)^7^, taken from the same property, with 8 months of age and 18.25 ± 7.23 kg average body weight were distributed in a randomized block design in a 3 × 3 factorial arrangement [(three levels of cactus pear silage—0, 21 and 42% total dry matter—DM) and three water supply intervals (0, 24 and 48 h)] and four animals per treatment. Animals with water supply intervals of 24 h received water on alternate days, while animals with water supply intervals of 48 h had water restriction for 48 h and received water for 24 h. The animals were housed in individual stalls (1.0 by 2.0 m), that was equipped with water troughs and feed troughs.

The experimental period lasted 75 days, preceded by 12 days for adaptation of the animals to the environment and the pens, and during this period all animals were treated against internal and external parasites with Ivermectin (Ivomec® Gold, Merial, Brazil) and vaccinated with a Clostridium vaccine (Sintoxan® Polivalente, Merial, Brazil), and orally supplemented with vitamins and minerals.

### Cactus pear silage

Cactus pear (*Opuntia ficus-indica* Mill.) was obtained from a single rural property, and all the cladodes, except for the main and primary ones, were collected, processed in a stationary forage machine to a particle size of 3 cm and ensiled in polyethylene drums with a capacity of 200 L. Silos were sealed with plastic tapes and lids to promote the fermentation process. The silage was used after a minimum period of 60 days after preparation.

Silage samples were collected at the time of supplying the diets and every 15 days for analysis of ammonia nitrogen (NH_3_-N)^41^, organic acids^42^ pH, DM recovery, and gas and effluent losses^43^. The silage presented the following fermentation characteristics: 3.81 pH; NH_3_-N of 9.0% total nitrogen; 59.20 g/kg DM and 22.80 kg/t green mass, respectively, for gas and effluent losses; 92.30% DM recovery and 80.2 g/kg DM lactic acid; 22.5 g/kg DM acetic acid; 0.5 g/kg DM butyric acid and 8.10 g/kg DM propionic acid^7^.

### Diets and general procedures

Diets were formulated to meet the nutritional requirements of crossbred goats with 18.5 kg and on estimated weight gain of 100 g/day following the National Research Council^44^ recommendations for daily intake of 96.63 g/day CP and 0.44 kg/day TDN. It consisted of Tifton 85 hay, cactus pear silage, ground corn, wheat bran, soybean meal and mineral premix. The forage: concentrate ratio was 60:40, and cactus pear silage was used to replace three proportions of Tifton 85 grass hay (0, 21 and 42% DM) in the diet (Tables 10 and 11). The diet was provided twice a day (09:00 and 15:00 h), and the amount of feed was adjusted daily with an acceptable amount of leftovers corresponding to 10% total amount provided to ensure ad libitum intake.

**Table 10:** Ingredients and chemical compositions of experimental diets (g/kg, DM basis).

Item	Cactus pear silage		
	0	21	42
**Ingredients**			
Ground corn	310	270	130
Soybean meal	70.0	90.0	90.0
Wheat bran	10.0	30.0	170
Tifton 85 hay	600	390	180
Cactus pear silage	0.00	210	420
Mineral premix^1^	10.0	10.0	10.0
**Chemical composition, g/kg DM**			
Dry matter, g/kg as fed	870	707	542
Ash	98.7	78.4	82.9
Crude protein	112	116	124
Ether extract	25.0	24.6	23.9
Neutral detergent fiber ap^2^	496	408	357
Neutral detergent insoluble nitrogen, g/kg CP	314	230	123
Acid detergent insoluble nitrogen, g/kg CP	78.0	63.0	42.9
Acid detergent fiber	263	225	196
Acid detergent lignin	55.3	50.3	46.9
Hemicellulose	233	183	161
Cellulose	208	175	149
Carbohydrate total	764	781	769
Non-fibrous carbohydrate	268	373	412
Metabolizable energy (Mcal/kg)	2.18	2.22	2.32
Total digestible nutrients	647	678	697
**Macrominerals, g/kg DM**			
Calcium, Ca	4.04	6.10	7.80
Phosphorus, P	2.44	2.44	2.44
Magnesium, Mg	3.21	3.47	3.70
Sodium, Na	3.75	4.51	5.56
Potassium, K	0.76	0.77	0.75
Ca:P ratio	1.65:1	2.50:1	3.48:1

^1^Mineral premix provided the guaranteed levels per kilogram of diet: KI 43 g, Ca 190 g, Mg 16 g, S 35 g, Cu 1540 mg, Fe 2190 mg, Zn 2170 mg, Mn 1335 mg.

^2^Neutral detergent fiber corrected for ash and protein.

**Table 11:** Fatty acid (g/100 g FAME) composition of experimental diets with cactus pear silage.

Fatty acid (g/100 g FAME)	Cactus pear silage		
	0	21	42
C12:0	0.32	0.35	0.35
C14:0	0.41	0.45	0.48
C16:0	19.1	19.2	19.5
C17:0	0.38	0.36	0.31
C18:0	2.16	2.17	2.26
C20:0	0.62	0.61	0.53
C22:0	0.43	0.38	0.35
C24:0	0.35	0.33	0.31
c9-C16:1	0.26	0.25	0.25
c9-C18:1	13.7	13.8	13.5
c11-C18:1	0.99	0.91	0.89
c12-C18:1	0.32	0.30	0.37
c9c12C18:2	44.0	44.0	42.9
C18:3 n-3	13.4	13.1	13.8
Other fatty acids	3.59	3.82	4.20

Samples of ingredients, leftovers and silage were collected weekly and frozen (− 20 °C) for further chemical analysis.

The methods proposed by AOAC^45^ were used to determine the concentrations of dry matter (DM, method 934.01), crude protein (CP, method 954.01), ether extract (EE, method 920.39), ash (method 942.05) and lignin (method 920.39). The content of acid detergent fiber (ADF) and NDF were quantified according to Van Soest^46^.

NDF was corrected for ash and protein (NDFap) according to Mertens^47^. Acid detergent insoluble nitrogen (ADIN) and neutral detergent insoluble nitrogen (NDIN) were measured using the recommendations of Licitra et al*.*^48^.

Non-fiber carbohydrates (NFC) and total carbohydrates (TC) were estimated according to the method described by Mertens^49^ and Sniffen et al*.*^50^, respectively.

Total digestible nutrients (TDN) and (ME) were calculated according to formulas proposed by the NRC^51^.

$$\begin{gathered} TDN = NFCdig + CPdig + (EEdig \times 2.25) + NDFdig - 7 \hfill \\ ME = (1.01 \times (DE - 0.45)) + 0.0046 \times (EE - 3) \hfill \\ \end{gathered}$$

where dig is digestible, and DE is digestible energy.

The content of calcium (Ca), phosphorus (P), magnesium (Mg), potassium (K) and sodium (Na) were determined in the ingredients on a dry matter basis, as described by Silva and Queiroz^52^.

Daily DM intake and gain averaged 650.67 g/day and 72.25 g/day, respectively, while total water intake (drinking + feeding), total water excretion (feces + urine), and body water balance/retention had averages of 2.36; 0.87 and 1.49, respectively^7^.

### Performance and carcass traits

At the end of the experiment, after the animals were subjected to 16 h fasting, slaughter weight (SW) and body condition score (BCS) were obtained by external palpation, considering the scales from 1 to 5, where 1 = very thin and 5 = very fat or obese, according to Cezar and Sousa^11^.

In the slaughter procedure, animals were slaughtered by cerebral concussion using a captive dart pistol (Tec 10 P model, CTrade Ltda, Porto Alegre, RS, Brazil) followed by bleeding by sectioning the carotid and jugular vessels, for three minutes. Thereafter, animals were skinned and eviscerated, and the gastrointestinal tract (GT) was weighed full and empty.

After removing the head, limb and tail, the carcass was weighed to obtain the hot carcass weight (HCW). The empty body weight (EBW) was calculated by the difference between the SW and the weight of the contents of the GT and the empty bladder and gallbladder.

A portable pH meter (Testo 205, Campinas, SP, Brazil) previously calibrated was used to measure the pH_initial_ (45 min after slaughter) and the pH_final_ (24 h after slaughter).

Carcasses were cooled for 24 h at ± 4 °C in a cold room. After this period, the carcasses were weighed to obtain the cold carcass weight (CCW) and water evaporation losses in cooling (WELC) according to Eq. (1). Subsequently, hot (HCY) and cold (CCY) and biological (BCY) yields were calculated according to Eqs. (2), (3) and (4) respectively.

1 $$WELC = \frac{HCW - CCW}{{HCW}} \times 100$$

2 $$HCY = \frac{HCW}{{SW}}$$

3 $$CCY = \frac{CCW}{{SW}}$$

4 $$BCY = \frac{EBW}{{SW}}$$

Carcass morphometric measurements were obtained according to Cezar and Sousa^11^: based on the parameters length (external, internal and leg), rump perimeter, chest width, and depth of chest. The carcass compactness index (CCI) was calculated by Eq. (5), expressed in kg/cm.

5 $${\text{CCI}} = \frac{CCW}{{{\text{internal length}}}}$$

Half carcasses were weighed and cut in five anatomical regions: shoulder (by disarticulating the scapula), leg (by the cut between the last lumbar vertebra and the first sacral vertebra), loin (between the first and the sixth lumbar vertebrae), ribs (between the first and the 13th thoracic vertebrae) and neck (region comprising the seven cervical vertebrae).

In the left half carcass, a cross section was made in the section between the 12th and 13th ribs to measure the loin eye area (LEA) of the *Longissimus thoracis* (LT) muscle and minimum subcutaneous fat thickness (Measure C), according to Cezar and Sousa^11^. The left *Longissimus lumborum* muscle were dissected, identified, packaged and frozen at − 18 °C for further analysis.

The left legs were dissected, and the leg muscularity index (LMI) was calculated using the weight in grams of the five muscles (W5M) that surround the femur (*Biceps femoris muscle, Semimembranosus muscle, Semitendinosus muscle, Adductor femoris muscle* and *Quadriceps femoris muscle*) and length of the femur (LF) in cm, using the Eq. (6) described by Purchas et al*.*^53^.

6 $$LMI = \frac{{\sqrt {W5M/LF} }}{LF}$$

### Physical–chemical composition of the *Longissimus lumborum* muscle

Two steaks approximately 2.5 cm thick were exposed to the atmosphere for 50 min to determine the color indices for each animal: L*, the index related to lightness (L* = 0, black; = 100, white); a*, the redness index that ranges from green (−) to red (+); and b*, the yellowness index that ranges from blue (−) to yellow (+); in three different locations, using a chromameter (MINOLTA CR-400, Osaka, Japan). The chromameter was calibrated with a white tile (Y = 93.2, x = 0.3137, y = 0.3257), Illuminant D-65, with 2° standard observer. The saturation index (*Chroma*) was determined according to the Eq. (7)

7 $$Chroma = \sqrt {a^{2} + b^{2} }$$

Cooking losses were obtained according to the methodology described by American Meat Science Association—AMSA^54^ and subsequently the shear force was measured. From the samples used in cooking losses (CL), at least three cylinders of each sample were removed, with a 1.27 cm diameter pourer, in the direction of muscle fibers. A texturometer equipped with a Warner–Bratzler stainless steel blade (G-R MANUFACTURING CO. 3000) with a 25 kgf load cell and a cutting speed of 20 cm/min was adopted, according to Wheeler et al*.*^55^.

The concentration of moisture, ash, and protein were obtained according to the recommendations of AOAC^56^. For lipid extraction, the method described by Hara and Radin^57^ was adopted, using n-hexane–isopropanol solution (3:2 v/v) and after extraction, lipids were esterified and methylated^58^.

### Fatty acid profile of the *Longissimus lumborum* muscle

To determine the fatty acid profile, the lipids previously extracted from the *Longissimus lumborum* muscle and diets (Table 2) were converted to fatty acid methyl esters (FAME). To analyze the fatty acids profile, trans methylated samples were analyzed in a gas chromatograph (Focus GC AI 3000, Thermo Finnigan analyzer, Milan, Italy) with a flame ionization detector and capillary column CP-Sil 88 (Varian) with 100 m length, 0.25 μm internal diameter and 0.20 μm film thickness.

Hydrogen was used as carrier gas, at a flow rate of 1.8 mL/min. The initial oven temperature program was 70 °C, with a waiting time of 4 min, 175 °C (13 °C/min), waiting time of 27 min, 215 °C (40 °C/min), waiting time of 9 min and then increasing 7 °C/min to 230 °C, remaining for 5 min, totaling 65 min. The vaporizer temperature was 250 °C and the detector temperature was 300 °C.

An aliquot of 1 μL of the esterified extract was injected into the chromatograph and the fatty acids (FA) were identified by comparing the retention times. The percentages of fatty acids were obtained using the Chromquest 4.1 software (Thermo Electron, Milan, Italy).

FAs were identified by comparing the retention times of methyl esters of the samples with FA standards (BCR-CRM 164, Anhydrous Milk-Fat Producer: BCR Institute for Reference Materials and Measurements; Supelco TM Component FAME Mix, cat 18919 Supelco, Bellafonte, PA). To quantify the FAME, a response factor was generated for each FA based on the standard sample. The results were quantified by normalizing the areas of methyl esters and expressed as mg/100 g fatty acid methyl esters (FAME). Only FAME representing > 0.01% of total FAME and in at least one of the treatments, were included in tables.

After FA identification, the sum of saturated fatty acids (SFA), branched chain fatty acids (BCFA), monounsaturated fatty acids (MUFA), polyunsaturated fatty acids (PUFA) and the MUFA/SFA, PUFA/SFA, PUFA/MUFA, n-6/n-3 ratios were calculated.

The nutritional quality of the lipid fraction was measured through the following indices: atherogenicity (AI) and thrombogenicity (TI) proposed by Ulbricht and Southgate^39^.

The ratio of hypocholesterolemic to hypercholesterolemic fatty acids (h:H) was calculated according to Santos-Silva et al*.*^40^ and the concentration of desirable fatty acids (DFA) according to Wood et al*.*^59^.

The activity of the enzymes Δ9-desaturase C16, Δ9-desaturase C18, and elongase was estimated according to De Smet et al*.*^60^.

### Statistical analysis

The experimental design was a randomized block design, blocked according to animal’ initial weight (n = 36), and the treatments were arranged in a 3 × 3 factorial, with three cactus pear silage inclusion levels (0%, 21% and 42% DM) and intermittent water supply (offers ad libitum, and 24 and 48 h water restriction) in the diets of goats, resulting in nine treatments and four replications per treatment.

Data were subjected to analysis of variance (ANOVA) and mean comparisons were done by Tukey’s test, as well as the interactions between them, with statistical probability of up to 5% using the PROC GLM of SAS 9.2 software, according to the Eq. (8):

8 $${\text{Yijk}} = {\upmu } + {\alpha i} + {\beta j} + ({{\upalpha \upbeta }}){\text{ij}} + {\text{k}} + {\text{eijk}}$$

where Y is the observed value of the variable ijk that refers to the k-th repetition of the combination of the i-th level of factor A with the j-th level of factor B, μ is the average of all experimental units for the variable, αi is the effect of the ratios of forage cactus silage (i = 0, 21, and 42%) at the observed value Yijk, βj is the effect of the intermittent water supply (j = 0, 24, and 48 h) at the observed value Yijk, αβij is the effect of the interaction between the ratio of forage cactus silage and intermittent water supply, k is the block effect on the observation Yijk, and eijk is the error associated with the observation of Yijk.

## Data Availability

The datasets used and/or analyzed during the current study available from the corresponding author on request.
